# Impact of Para-Aortic Lymphadenectomy on Clinically FIGO Stage IIIC1 High-Grade Endometrial Cancer: A Retrospective Cohort Study from Two Tertiary Centers in Korea and Taiwan

**DOI:** 10.3390/medicina61061079

**Published:** 2025-06-12

**Authors:** Yen-Ling Lai, Jun-Hyeong Seo, Koping Chang, Hyun-Soo Kim, Jung Chen, Tyan-Shin Yang, Yu-Li Chen, Yoo-Young Lee

**Affiliations:** 1 Department of Obstetrics and Gynecology, National Taiwan University Hospital Hsin-Chu Branch, Hsinchu 300195, Taiwan; adina@kimo.com; 2 Department of Obstetrics and Gynecology, National Taiwan University Hospital, College of Medicine, National Taiwan University, Taipei 100233, Taiwan; jjchen915@gmail.com (J.C.); jessica0617xyz@gmail.com (T.-S.Y.); 3 Division of Gynecologic oncology, Department of Obstetrics and Gynecology, Samsung Medical Center, School of Medicine, Sungkyunkwan University, Seoul 06351, Republic of Korea; junhyeong.seo@samsung.com; 4 Department of Pathology, National Taiwan University Hospital, Taipei 100225, Taiwan; koping@ntuh.gov.tw; 5 Department of Pathology and Translational Genomics, Samsung Medical Center, Sungkyunkwan University School of Medicine, Seoul 06351, Republic of Korea; hyun-soo.kim@samsung.com; 6 Department of Obstetrics and Gynecology, National Taiwan University Hospital Yun-Lin Branch, Yunlinounty 640203, Taiwan

**Keywords:** endometrial cancer, FIGO stage IIIC1, lymphadenectomy

## Abstract

*Background and Objectives*: The therapeutic effect of para-aortic lymphadenectomy in patients with clinically para-aortic node-negative diseases remains controversial. In this study, we investigated whether combined pelvic and para-aortic lymphadenectomy has a survival benefit compared with pelvic lymphadenectomy alone in patients with clinically FIGO stage IIIC1 high-grade endometrial cancer. *Materials and Methods*: We retrospectively reviewed patients with clinically FIGO stage IIIC1 high-grade endometrial cancer in the period between January 2000 and December 2020 at two tertiary centers. The patients were stratified according to type of lymphadenectomy and subgroup analyses performed. Kaplan–Meier analysis and a Cox proportional-hazards model were used to evaluate survival outcomes. *Results*: A total of 56 patients were identified. Of these patients, 18 underwent pelvic lymphadenectomy alone and 38 underwent combined pelvic and para-aortic lymphadenectomy. After staging surgery, 34 (60.7%) patients had pathologically confirmed lymph node metastases. Within a median follow-up of 57.5 months, there were no significant differences in recurrence-free survival (RFS) and overall survival (OS) between the two groups. In subgroup analyses, the node- and lymphovascular space invasion (LVSI)-positive patients characterized by grade 3 endometrioid histologic type (*p* = 0.010) or negative peritoneal washing cytology (*p* = 0.035) had an RFS benefit from combined pelvic and para-aortic lymphadenectomy. *Conclusions*: The addition of para-aortic lymphadenectomy to pelvic lymphadenectomy did not improve survival in patients with clinically FIGO IIIC1 endometrial cancer. However, para-aortic lymphadenectomy may have RFS benefit for patients with grade 3 endometrioid histologic type and positive LVSI.

## 1. Introduction

Endometrial cancer is the most common gynecologic cancer among women, with increasing incidence rates worldwide [1]. In addition, uterine cancer is one of the few cancers with increasing mortality, probably due to a lack of major treatment advances [1,2]. The increasing mortality associated with endometrial cancer highlights the growing clinical urgency surrounding its management. This includes the need to optimize surgical procedures, particularly the role of lymphadenectomy in improving staging accuracy and potentially influencing outcome, especially in high-risk subgroups. Approximately 30% of endometrial cancer is diagnosed as a locally advanced disease or with distant metastases. Of these advanced cases, stage IIIC disease accounts for 8% of endometrial cancer diagnoses [3]. The FIGO (the International Federation of Gynecology and Obstetrics) staging system subdivides locoregional nodal metastasis into IIIC1 (metastases to the pelvic lymph nodes) and IIIC2 (metastases to the para-aortic lymph nodes) disease.

Surgical staging has been the primary treatment approach for patients with endometrial cancer [4]. Systemic lymphadenectomy, including pelvic lymphadenectomy and para-aortic lymphadenectomy, is regarded as an integral part of staging surgeries, especially in patients with high-risk endometrial cancer. It can provide diagnostic value for determining the optimal adjuvant therapy. However, the therapeutic significance of lymphadenectomy for clinically node-negative disease remains unclear [5,6].

The SEPAL study, which compared the survival benefits of combined pelvic and para-aortic lymphadenectomy with those of pelvic lymphadenectomy, found that combined pelvic and para-aortic lymphadenectomy had survival benefit for patients with endometrial cancer of intermediate or high risk of recurrence [7]. However, the SEPAL study did not exclude patients with preoperatively identified para-aortic lymphadenopathy. Therefore, it could not be ruled out that the observed survival benefit of para-aortic lymphadenectomy in the SEPAL study might have been due to the therapeutic effect of debulking. To further investigate whether para-aortic lymphadenopathy confers a survival benefit in patients with high-grade, early-stage disease, we examined the impact of combined systematic pelvic and para-aortic lymphadenectomy. Our findings indicated that this combined approach did not improve survival compared with pelvic lymphadenectomy alone [8]. However, in clinical practice, when preoperative imaging demonstrates pelvic lymphadenopathy without evidence of para-aortic lymph node involvement, the survival benefit of performing para-aortic lymphadenectomy remains unclear. Therefore, we performed this retrospective cohort study from two tertiary centers in Korea and Taiwan to investigate whether combined pelvic and para-aortic lymphadenectomy in comparison with pelvic lymphadenectomy alone provides a survival benefit in patients with clinically stage IIIC1 endometrial cancer.

## 2. Materials and Methods

This is a retrospective cohort study. Patients with clinically FIGO stage IIIC1 high-grade endometrial cancer undergoing staging surgeries in the period between January 2000 and December 2020 at Samsung Medical Center (SMC) in Seoul, Korea, and National Taiwan University Hospital (NTUH) in Taipei, Taiwan were included. The study protocol was approved by the institutional review boards of the two centers. The requirement for informed consent was waived by the ethics committee.

The staging surgery included total hysterectomy, bilateral salpingo-oophorectomy, pelvic lymphadenectomy with or without para-aortic lymphadenectomy, omental biopsy, and peritoneal washing cytology. Any identified suspicious lesions were excised. During pelvic lymphadenectomy, nodes located in the external iliac, internal iliac, obturator, and common iliac regions were excised. As for the para-aortic lymph nodes, they were situated around the vena cava (right para-aortic), between the vena cava and aorta (middle), and on the left side of the aorta (left para-aortic). The common cephalad border of para-aortic lymphadenectomy was at least to the level of the inferior mesenteric artery. Some gynecologic oncologists performed para-aortic lymphadenectomy to the level of the renal vein to comprehensively evaluate the status of the para-aortic lymph nodes. The staging surgery was performed by laparoscopy (conventional or robotic) or laparotomy.

At the two institutions, patients diagnosed with endometrial cancer through biopsy preoperatively underwent image screening (magnetic resonance imaging [MRI] or computed tomography [CT]) to identify potential lymph node or distant metastases. According to 2023 FIGO staging for endometrial cancer [9], stage IIIC is defined by tumor metastasis to pelvic or para-aortic lymph nodes or both. Stage IIIC is further divided into IIIC1 (metastasis to the pelvic lymph nodes) and IIIC2 (metastasis to para-aortic lymph nodes up to the renal vessels, with or without metastasis to the pelvic lymph nodes). This study focused on patients clinically staged as IIIC1, meaning those who had pelvic lymphadenopathy identified on preoperative imaging without evidence of para-aortic lymphadenopathy or extrauterine metastases. When the gynecologic oncologists thought the survival benefit of removing microscopic para-aortic metastases outweighed adverse effects of lymphadenectomy or medical comorbidities of patients, they performed para-aortic lymphadenectomy. Therefore, some patients were subsequently upstaged to IIIC2 or even stage IV based on postoperative pathological findings. Medical comorbidities mainly included obesity and functional impairment of major organs such as heart, lung, liver, and kidney.

We reviewed the clinical and pathological characteristics of the study population from medical records, including age at initial disease diagnosis, body mass index (BMI), pre-operative serum levels of cancer antigen 125 (CA-125), FIGO stage of disease, surgical methods, tumor histologic types, depth of the myometrial invasion, status of lymphovascular space invasion (LVSI), results of the peritoneal washing cytology, and types of adjuvant treatment. Adjuvant treatment for endometrial cancer included chemotherapy, radiotherapy, or combined chemotherapy and radiotherapy.

After the primary treatment was completed, regular surveillance was arranged every 3 months for 3 years, and then every 6 months thereafter. A smear of the vaginal cuff was performed every 6 months. CT or MRI was arranged based on patient symptoms and clinical concern for disease relapse or metastasis. Recurrence was defined according to RECIST 1.1 for image studies or biopsy-proven disease. Recurrence-free survival (RFS) and overall survival (OS) were investigated for patients who underwent para-aortic lymphadenectomy compared with those who did not. RFS was defined as the interval from the date of surgery to the date of first recurrence or last follow-up. OS was calculated as the interval from the date of surgery to the date of disease-related death or last follow-up.

All results were presented as frequency and rate for categorical variables or median and range for continuous variables. Categorical variables were evaluated by the chi-square test, and continuous variables were compared by the Mann–Whitney U test. Survival rates were estimated by Kaplan–Meier analysis. The log-rank test was used to compare survival between groups. *p* < 0.05 was considered significant. A Cox proportional-hazards regression model was used to assess the independent effects of different clinicopathological factors on survival. Covariates reaching a threshold significance of *p* < 0.05 on univariable analysis were determined for the analysis of multivariable models. Statistical assessment was performed with MedCalc software 14.12.0 (MedCalc Software bvba, Ostend, Belgium).

## 3. Results

### 3.1. Characteristics of the Study Population

A total of 56 patients with clinically FIGO stage IIIC1 high-grade endometrial cancer were identified during the study period. Of these patients, 18 (32.1%) underwent pelvic lymphadenectomy alone and 38 (67.9%) underwent combined pelvic and para-aortic lymphadenectomy. The median follow-up duration from the initial disease diagnosis until the date of death or last contact was 57.5 months (range, 2.0–146.0 months).

Table 1 shows the clinical and pathological characteristics of the 56 eligible patients based on the types of lymphadenectomy. No significant differences were found in the distribution of the variables between the two groups. The median age at diagnosis of endometrial cancer was 59.5 years (range, 31.0–82.0 years), and the median BMI of patients was 26.6 kg/m^2^ (range, 16.3–34.6 kg/m^2^). The median pre-operative serum CA-125 level was 145.5 U/mL (range, 4.8–944.3 U/mL). Among these 56 patients, 45 (80.4%) received laparotomy and 11 (19.6%) received laparoscopic surgeries.

Grade 3 endometrioid carcinoma (N = 28, 50.0%) was the most common histologic subtype, followed by serous (N = 7, 12.5%) and mixed type carcinoma (N = 7, 12.5%). The majority of tumors were characterized by ≥1/2 depth of myometrial invasion (N = 40, 71.4%), presence of LVSI (N = 39, 69.6%) and negative peritoneal washing cytology (N = 43, 76.8%). Eighteen of 56 patients (32.1%) were pathologically confirmed with early-stage (FIGO stage I–II) disease. Among the remaining 38 cases (67.9%) with advanced tumors (FIGO III–IV), 2 had stage IIIA disease, 1 had stage IIIB disease, 26 had stage IIIC1 disease, 8 had stage IIIC2 disease, and 1 had stage IVA disease. The stage IVA disease was diagnosed with mucosal involvement of bowel but without lymph node involvement. Adjuvant treatment was administered to 53 (94.6%) patients, most of whom underwent combination treatment (N = 26, 46.4%).

### 3.2. Analyses of Factors Affecting Survival in Different Subgroups

Table 2 demonstrates the results of Cox regression analysis for the 56 patients with clinically FIGO stage IIIC1 high-grade endometrial cancer. In univariable analysis of factors associated with RFS, advanced FIGO stage (*p* = 0.012) and presence of LVSI (*p* = 0.013) were associated with shorter RFS. In multivariable analysis, only advanced FIGO stage (hazard ratio [HR]: 8.65, 95% confidence interval (CI) 1.15–65.32, *p* = 0.037) was significantly associated with poor RFS. In univariable analysis of factors associated with OS, the significant variable affecting OS was advanced FIGO stage (HR: 3.38, 95% CI 1.79–14.48, *p* = 0.040).

Of the 56 patients with clinically FIGO stage IIIC1 high-grade endometrial cancer, 34 had pathologically confirmed lymph node involvement. The prognostic factors associated with the survival of the 34 patients are further evaluated in Table 3. In univariable analysis of factors associated with RFS, non-endometrioid histologic types (*p* = 0.016) and positive peritoneal washing cytology (*p* = 0.021) were associated with shorter RFS. Administration of adjuvant treatment with combined modalities was associated with longer RFS (*p* = 0.018). In multivariable analysis, the presence of non-endometrioid histologic types (HR: 3.34, 95% CI 1.11–10.05, *p* = 0.032) was an independent factor contributing to the risk of disease recurrence. In univariable analysis of factors associated with OS, the only significant variable affecting OS was positive peritoneal washing cytology (HR: 59.88, 95% CI 4.48–800.24, *p* = 0.002).

LVSI, another negative prognostic factor (Table 2), could be detected in 28 of the 34 cases (82.4%) with pathologically confirmed lymph node involvement. Table 4 shows the results of prognostic analysis for these 28 patients. In a univariable analysis of factors associated with RFS, the presence of non-endometrioid histologic types (*p* = 0.025) was associated with shorter RFS. The performance of combined pelvic and para-aortic lymphadenectomy was associated with longer RFS (*p* = 0.042). In a multivariable analysis, the presence of non-endometrioid histologic types (HR: 3.44, 95% CI 1.05–11.26, *p* = 0.041) was the factor impacting RFS. In a univariable analysis of factors associated with OS, the only significant variable affecting OS was positive peritoneal washing cytology (HR: 25.38, 95% CI 2.34–274.91, *p* = 0.008).

### 3.3. Survival Impact of Para-Aortic Lymphadenectomy on Patients with Pathologically Confirmed Nodal Involvement and Positive LVSI

**Figure 1:**
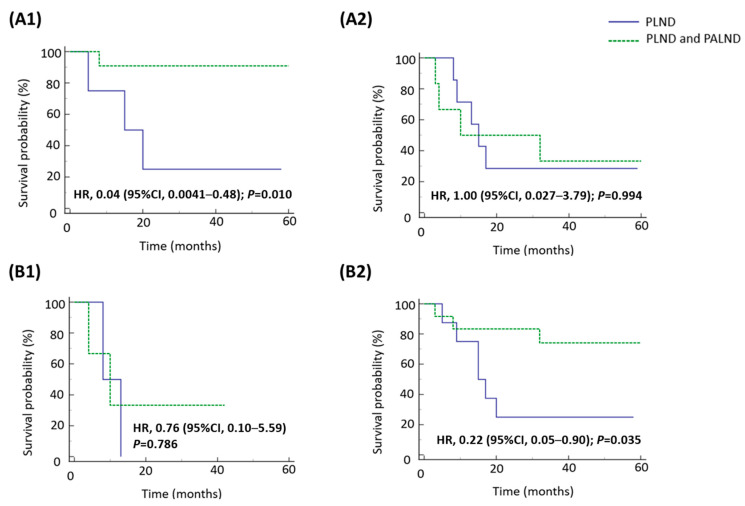
Kaplan–Meier analysis of recurrence-free survival for patients with pathologically confirmed lymph node involvement and LVSI according to type of lymphadenectomy in different subgroups. (**A1**) Grade 3 endometrioid, (**A2**) non-endometrioid, (**B1**) cytology positive, and (**B2**) cytology negative.

**Figure 2:**
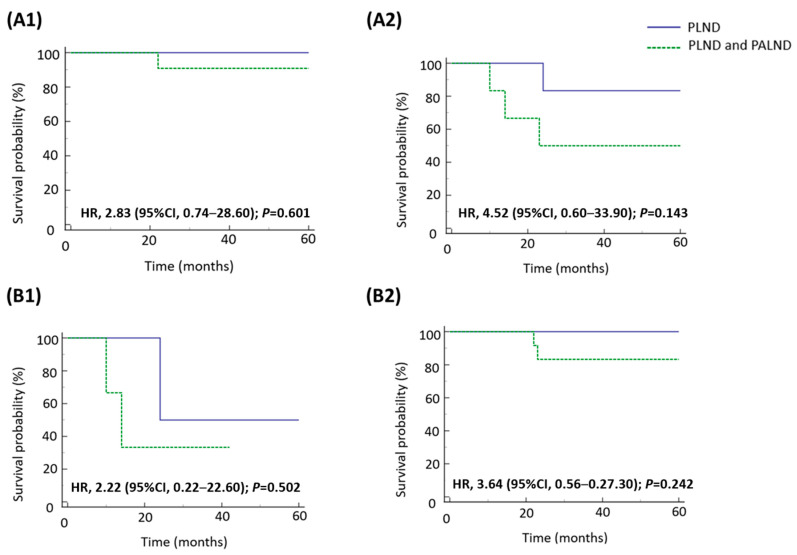
Kaplan–Meier analysis of overall survival for patients with pathologically confirmed lymph node involvement and LVSI according to type of lymphadenectomy in different subgroups. (**A1**) Grade 3 endometrioid, (**A2**) non-endometrioid, (**B1**) cytology positive, and (**B2**) Cytology negative.

The addition of para-aortic lymphadenectomy did not improve the survival of the 28 patients with pathologically confirmed nodal involvement and positive LVSI (Table 4). However, an RFS benefit was observed in patients with grade 3 endometrioid and negative peritoneal washing cytology in subgroup analyses. These two subgroups receiving combined pelvic and para-aortic lymphadenectomy had better RFS compared with those receiving pelvic lymphadenectomy alone (Figure 1). Combined pelvic and para-aortic lymphadenectomy was not associated with an OS benefit from individual variables in patients with pathologically confirmed nodal involvement and LVSI (Figure 2).

## 4. Discussion

In this retrospective study, patients with clinically FIGO stage IIIC1 high-grade endometrial cancer postoperatively showed an overall risk of 60.7% of lymph node metastases. We found that the pathological disease stage was associated with patient survival in the study population. The addition of para-aortic lymphadenectomy to pelvic lymphadenectomy did not improve RFS or OS, compared with pelvic lymphadenectomy alone. However, in our subgroup analysis, the node- and LVSI-positive patients characterized by grade 3 endometrioid histologic type or negative peritoneal washing cytology had an RFS benefit from combined pelvic and para-aortic lymphadenectomy.

The role of lymphadenectomy for patients with endometrial cancer has long been a subject of great debate [10,11]. Many retrospective analyses have suggested that there are survival benefits of systemic pelvic and para-aortic lymphadenectomy in patients with endometrial cancer [7,12,13,14], and, accordingly, many patients have undergone this procedure over the years. However, there are several pitfalls that could influence the impact of para-aortic lymphadenectomy in these studies. First, the eligibility criteria were not strict. Many of them include all endometrial cancer patients, including those with early-stage and those with advanced-stage disease. In addition, patients with enlarged lymph nodes were not excluded [7,13]. The removal of palpable lymph nodes may achieve optimal cytoreduction, potentially resulting in improved patient survival. Second, patients in different lymphadenectomy groups did not receive uniform adjuvant treatment in some studies. This inconsistency might be a confounding factor for survival outcomes. Third, para-aortic lymphadenectomy is a procedure with substantial treatment-related challenges. Surgeons decide whether to perform this procedure not only according to disease-specific factors such as disease stage, histologic types, thickness of myometrial invasion, and LVSI status, but also on the basis of patient age, performance status, and comorbidities. Individuals with poorer performance status might not receive para-aortic lymphadenectomy, while younger and healthier patients are more likely to undergo systematic pelvic and para-aortic lymphadenectomy.

For clinically node-negative patients, systemic pelvic and para-aortic lymphadenectomy provides accurate disease stage, which can guide observation or appropriate adjuvant therapy [15]. However, the therapeutic significance of systemic lymphadenectomy in patients with clinically node-negative disease remains unclear [15,16]. In our previous study, combined pelvic and para-aortic lymphadenectomy compared with pelvic lymphadenectomy alone did not improve survival in patients with pathologically diagnosed early-stage high-grade endometrial cancers [8]. In the further analysis of patients with clinically early-stage disease, although 30.3% of these patients were pathologically proven to have lymph node metastases after staging surgeries, no significant differences in the RFS and OS were noted between the two lymphadenectomy groups.

In our current findings, the addition of para-aortic lymphadenectomy to pelvic lymphadenectomy also did not improve the RFS or OS of patients with clinically FIGO stage IIIC1 high-grade endometrial cancer, compared with the RFS or OS after pelvic lymphadenectomy alone. Based on our previous and current findings, patients with clinically para-aortic node-negative high-grade endometrial cancer, the surgical staging procedure with pelvic lymphadenectomy alone seems sufficient. However, in our subgroup analysis, the node- and LVSI-positive patients characterized by grade 3 endometrioid histologic type or negative peritoneal washing cytology had an RFS benefit from combined pelvic and para-aortic lymphadenectomy. The survival benefit of para-aortic lymphadenectomy in patients with LVSI-positive and grade 3 endometrioid carcinoma might be attributable to the inclusion of a subgroup with particularly poor prognosis, such as those harboring p53 mutations. This is supported by the fact that grade 3 endometrioid carcinoma represents a prognostically, clinically, and molecularly heterogeneous disease [9]. All these patients underwent uniform postoperative treatment with chemotherapy and radiotherapy. Therefore, preoperative identification of this subpopulation is essential. However, these findings were based on relatively small sample sizes and were exploratory in nature. As such, the observed RFS benefit in these subgroups should be interpreted with caution. Although the current data provide preliminary observations that suggest a potential benefit in certain subgroups, we acknowledge that these findings were not sufficient to support definitive clinical recommendations.

Like this current study, several studies have established that LVSI, which can be obtained from endometrial biopsy specimens, is highly associated with nodal metastases in endometrial cancer [17,18,19]. In our study, 71.8% of patients (28/39) with positive LVSI had nodal metastase. Twenty of twenty-eight patients (71.4%) with positive LVSI had negative peritoneal washing cytology. Therefore, histologic type and LVSI status from preoperative specimens may assist surgeons in deciding whether to perform para-aortic lymphadenectomy for the patients with clinically FIGO stage IIIC1 high-grade endometrial cancer.

In addition, several preoperative scoring systems have been proposed and validated to predict the risk of nodal metastasis in endometrial cancer patients [20,21,22,23,24]. The scoring systems can be based on risk factors that can be assessed preoperatively, such as tumor histologic type/grade, serum CA-125 level, depth of myometrial invasion, tumor size, and the presence or absence of cervical involvement [20,21,22,25]. Because some molecular subtypes of endometrial cancer are significantly associated with lymph node metastasis—the P53-abnormal group demonstrates the highest incidence of nodal involvement, whereas the POLE-mutated group has the lowest [26,27]—such molecular classification has become a promising factor with which to tailor surgical treatment.

The limitations of this study include its retrospective study design and the relatively small number of studied patients. The inherent limitations associated with the retrospective design may affect the results of our findings, particularly in interpreting causal relationships between lymphadenectomy and survival outcomes. Our results should be interpreted as hypothesis-generating and warrant further investigation in prospective studies to confirm the effects. Furthermore, selection bias may have arisen if clinical assessments regarding the extent of lymphadenopathy were influenced by inter-observer variability among radiologists interpreting the preoperative imaging studies. In addition, we lack information on the molecular classification of our patients. However, this study benefited from a well-defined study population with clinically FIGO stage IIIC1 high-grade endometrial cancer. Furthermore, there were no significant differences observed in the distribution of clinical and pathological variables, surgical procedures, and adjuvant therapeutic approaches between the two lymphadenectomy cohorts.

## 5. Conclusions

In conclusion, the present study demonstrated that the addition of para-aortic lymphadenectomy to pelvic lymphadenectomy did not improve survival in patients with clinically FIGO IIIC1 endometrial cancer. Performance of para-aortic lymphadenectomy may improve RFS in node- and LVSI-positive patients characterized by grade 3 endometrioid histologic type or negative washing cytology. Thus, further randomized controlled trials with molecular information are needed to validate the role of lymphadenectomy in endometrial cancer. These would help patients with endometrial cancer receive the most appropriate surgical and adjuvant treatment, thereby reducing lymphadenectomy-related complications. However, until more evidence is available, our research still holds reference value that for patients with clinically stage IIIC1 enometrial cancer, para-aortic lymphadenectomy may be considered in those with grade 3 endometrioid histologic type and positive LVSI.

## Figures and Tables

**Table 1:** Clinical and pathological characteristics of study population (n = 56).

	PLND (n = 18)	PLND and PALND (n = 38)	Total (n = 56)	*p* Value ^a^
**Age (median, range)**	64.0 (33.0–78.0)	58.0 (31.0–82.0)	59.5 (31.0–82.0)	0.642
<60 years	8 (44.4%)	28 (73.7%)	36 (64.3%)	
≥60 years	10 (55.6%)	10 (26.3%)	20 (35.7%)	
**BMI (median, range)**	23.0 (20.4–29.7)	25.7 (16.3–34.6)	26.6 (16.3–34.6)	0.313
**Pre-operative CA-125 (U/mL)** **(median, range)**	176.2 (4.8–449.4)	41.3 (4.8–944.3)	145.5 (4.8–944.3)	0.563
**Surgical method**				0.296
Laparotomy	13 (72.2%)	32 (84.2%)	45 (80.4%)	
Laparoscopy ^b^	5 (27.8%)	6 (15.8%)	11 (19.6%)	
**Histology**				0.257 ^c^
Grade 3 endometrioid	7 (38.9%)	21 (55.3%)	28 (50.0%)	
Non-endometrioid	11 (61.1%)	17 (44.7%)	28 (50.0%)	
Serous	4 (22.2%)	3 (7.9%)	7 (12.5%)	
Clear cell	1 (5.6%)	2 (5.3%)	3 (5.4%)	
Mixed type	2 (11.1%)	5 (13.1%)	7 (12.5%)	
Others ^d^	4 (22.2%)	7 (18.4%)	11 (19.6%)	
**Pathologic FIGO stage** ^e^				0.278 ^f^
I	1 (5.6%)	9 (23.7%)	10 (17.8%)	
II	3 (16.6%)	5 (13.2%)	8 (14.3%)	
IIIA	1 (5.6%)	1 (2.6%)	2 (3.6%)	
IIIB	0 (0.0%)	1 (2.6%)	1 (1.8%)	
IIIC1	13 (72.2%)	13 (34.2%)	26 (46.4%)	
IIIC2	0 (0.0%)	8 (21.1%)	8 (14.3%)	
IVA	0 (0.0%)	1 (2.6%)	1 (1.8%)	
**Myometrial invasion**				0.179
<1/2	3 (16.7%)	13 (34.2%)	16 (28.6%)	
≥1/2	15 (83.3%)	25 (65.8%)	40 (71.4%)	
**LVSI**				0.741
Present	12 (66.7%)	27 (71.1%)	39 (69.6%)	
Absent	6 (33.3%)	11 (28.9%)	17 (30.4%)	
**Peritoneal washing cytology**				0.426
Positive	2 (11.1%)	3 (7.9%)	5 (8.9%)	
Negative	15 (83.3%)	28 (73.7%)	43 (76.8%)	
Unknown	1 (5.6%)	7 (18.4%)	8 (14.3%)	
**Adjuvant treatment**				0.349
None	1 (5.6%)	2 (5.2%)	3 (5.4%)	
Chemotherapy	4 (22.2%)	9 (23.7%)	13 (23.2%)	
Radiotherapy ^g^	2 (11.1%)	12 (31.6%)	14 (25.0%)	
Combination treatment	11 (61.1%)	15 (39.5%)	26 (46.4%)	

PLND: pelvic lymphadenectomy; PALND: para-aortic lymphadenectomy; n: number; BMI: body mass index; FIGO: International Federation of Gynecology and Obstetrics; CA-125: cancer antigen 125; LVSI: lymph-vascular space invasion. ^a^ *p*-values of age, BMI, and pre-operative CA-125 were determined by Mann–Whitney U test and the others were determined by Chi-squared test. ^b^ Conventional laparoscopic and robotic surgeries were included. ^c^ Statistical analysis was performed for grade 3 endometrioid adenocarcinoma versus non-endometrioid carcinoma (including serous, clear cell, mixed type and other types of carcinoma). ^d^ Glassy cell carcinoma, mullerian carcinofibroma, villoglandular adenocarcinoma, squamous cell carcinoma, mesonephric carcinoma, choriocarcinoma, and high-grade carcinoma were included. ^e^ The disease stage was based on the 2009 FIGO staging system. ^f^ Statistical analysis was performed for FIGO stage I–II versus stage III–IVA endometrioid cancer. ^g^ Radiotherapy included external beam radiotherapy (EBRT) alone, vaginal brachytherapy alone, or EBRT plus vaginal brachytherapy.

**Table 2:** Univariable and multivariable analysis of prognostic factors for RFS and OS by Cox regression model in study population (n = 56).

	RFS				OS			
Variables	Univariable		Multivariable		Univariable		Multivariable	
	HR (95% CI)	*p* Value	HR (95% CI)	*p* Value	HR (95% CI)	*p* Value	HR (95% CI)	*p* Value
**Age**								
<60 years	1.00				1.00			
≥60 years	1.74 (0.64–4.76)	0.278			0.71 (0.15–3.33)	0.665		
**FIGO stage**								
I-II	1.00		1.00		1.00			
III-IVA	3.65 (1.34–9.95)	0.012	8.65 (1.15–65.32)	0.037	3.38 (1.79–14.48)	0.040		
**Histology**								
Grade 3 endometrioid	1.00				1.00			
Non-endometrioid	2.21 (0.85–5.77)	0.105			2.58 (0.58–11.37)	0.211		
**Surgical method**								
Laparotomy	1.00				1.00			
Laparoscopy	0.57 (0.18–1.82)	0.341			0.75 (0.11–4.99)	0.763		
**Extent of lymphadenectomy**								
PLND	1.00				1.00			
PLND and PALND	0.45 (0.16–1.27)	0.129			2.10 (0.40–10.89)	0.379		
**LVSI**								
Absent	1.00				1.00			
Present	3.59 (1.31–9.84)	0.013			1.97 (0.37–10.49)	0.426		

RFS: recurrence-free survival; OS: overall survival; n: number; HR: hazard ratio; CI: confidence interval; FIGO: International Federation of Gynecology and Obstetrics; PLND: pelvic lymphadenectomy; PALND: para-aortic lymphadenectomy; LVSI: lymph-vascular space invasion.

**Table 3:** Univariable and multivariable analysis of prognostic factors for RFS and OS by Cox regression model in patients with pathologic stage IIIC1 and IIIC2 endometrial cancer (n = 34).

	RFS				OS			
Variables	Univariable		Multivariable		Univariable		Multivariable	
	HR (95% CI)	*p* Value	HR (95% CI)	*p* Value	HR (95% CI)	*p* Value	HR (95% CI)	*p* Value
**Age**								
<60 years	1.00				1.00			
≥60 years	1.82 (0.58–5.74)	0.308			0.92 (0.18–4.66)	0.92		
**Histology**								
Grade 3 endometrioid	1.00		1.00		1.00			
Non-endometrioid	3.98 (1.30–12.17)	0.016	3.34 (1.11–10.05)	0.032	4.03 (0.88–18.53)	0.073		
**Surgical method**								
Laparotomy	1.00				1.00			
Laparoscopy	0.65 (0.18–2.33)	0.505			0.89 (0.11–7.12)	0.915		
**Extent of lymphadenectomy**								
PLND	1.00				1.00			
PLND and PALND	0.36 (0.12–1.09)	0.07			2.50 (0.50–12.38)	0.263		
**LVSI**								
Absent	1.00				1.00			
Present	2.40 (0.67–8.60)	0.179			1.65 (0.28–9.75)	0.583		
**Peritoneal washing cytology**								
Negative	1.00				1.00			
Positive	7.90 (1.36–45.87)	0.021			59.88 (4.48–800.24)	0.002		
**Adjuvant treatment**								
Single treatment	1.00				1.00			
Combination treatment	0.24 (0.08–0.78)	0.018			0.51 (0.11–2.37)	0.387		

RFS: recurrence-free survival; OS: overall survival; n: number; HR: hazard ratio; CI: confidence interval; PLND: pelvic lymphadenectomy; PALND: para-aortic lymphadenectomy; LVSI: lymph-vascular space invasion.

**Table 4:** Univariable and multivariable analysis of prognostic factors for RFS and OS by Cox regression model in patients with pathologic stage IIIC1 and IIIC2, LVSI-positive endometrial cancer (n = 28).

	RFS				OS			
Variables	Univariable		Multivariable		Univariable		Multivariable	
	HR (95% CI)	*p* Value	HR (95% CI)	*p* Value	HR (95% CI)	*p* Value	HR (95% CI)	*p* Value
**Age**								
<60 years	1.00				1.00			
≥60 years	1.44 (0.44–4.73)	0.548			0.55 (0.0.9–3.32)	0.55		
**Histology**								
Grade 3 endometrioid	1.00		1.00		1.00			
Non-endometrioid	3.64 (1.18–11.26)	0.025	3.44 (1.05–11.26)	0.041	4.86 (0.97–24.37)	0.054		
**Surgical method**								
Laparotomy	1.00				1.00			
Laparoscopy	1.24 (0.24–6.34)	0.796			1.91 (0.14–26.37)	0.629		
**Extent of lymphadenectomy**								
PLND	1.00				1.00			
PLND and PALND	0.30 (0.09–0.96)	0.042			2.38 (0.43–13.08)	0.319		
**Peritoneal washing cytology**								
Negative	1.00				1.00			
Positive	5.04 (0.97–26.10)	0.054			25.38 (2.34–274.91)	0.008		

RFS: recurrence-free survival; OS: overall survival; LVSI: lymph-vascular space invasion; n: number; HR: hazard ratio; CI: confidence interval; PLND: pelvic lymphadenectomy; PALND: para-aortic lymphadenectomy.

## Data Availability

The data that support the findings of this study are available from the corresponding author upon reasonable request.

## Abbreviations

The following abbreviations are used in this manuscript:

FIGO	International Federation of Gynecology and Obstetrics
RFS	Recurrence-free survival
OS	Overall survival
HR	Hazard ratio
CI	Confidence interval
LVSI	Lymph-vascular space invasion
SMC	Samsung Medical Center
NTUH	National Taiwan University Hospital
MRI	Magnetic resonance imaging
CT	Computed tomography
BMI	Body mass index
CA-125	Cancer antigen 125
PLND	Pelvic lymphadenectomy
PALND	Para-aortic lymphadenectomy
